# Inhibition of tyrosine kinase Fgr prevents radiation-induced pulmonary fibrosis (RIPF)

**DOI:** 10.1038/s41420-023-01538-3

**Published:** 2023-07-17

**Authors:** Amitava Mukherjee, Michael W. Epperly, Renee Fisher, Wen Hou, Donna Shields, M. Saiful Huq, Phillip M. Pifer, Ria Mulherkar, Tyler J. Wilhite, Hong Wang, Peter Wipf, Joel S. Greenberger

**Affiliations:** 1 Department of Radiation Oncology, UPMC Hillman Cancer Center, Pittsburgh, PA 15232 USA; 2 Department of Biostatistics, University of Pittsburgh, Pittsburgh, PA 15260 USA; 3 Department of Chemistry, University of Pittsburgh, Pittsburgh, PA 15260 USA

**Keywords:** Molecular modelling, Cell migration

## Abstract

Cellular senescence is involved in the development of pulmonary fibrosis as well as in lung tissue repair and regeneration. Therefore, a strategy of removal of senescent cells by senolytic drugs may not produce the desired therapeutic result. Previously we reported that tyrosine kinase Fgr is upregulated in ionizing irradiation-induced senescent cells. Inhibition of Fgr reduces the production of profibrotic proteins by radiation-induced senescent cells in vitro; however, a mechanistic relationship between senescent cells and radiation-induced pulmonary fibrosis (RIPF) has not been established. We now report that senescent cells from the lungs of mice with RIPF, release profibrotic proteins for target cells and secrete chemotactic proteins for marrow cells. The Fgr inhibitor TL02-59, reduces this release of profibrotic chemokines from the lungs of RIPF mice, without reducing numbers of senescent cells. In vitro studies demonstrated that TL02-59 abrogates the upregulation of profibrotic genes in target cells in transwell cultures. Also, protein arrays using lung fibroblasts demonstrated that TL02-59 inhibits the production of chemokines involved in the migration of macrophages to the lung. In thoracic-irradiated mice, TL02-59 prevents RIPF, significantly reduces levels of expression of fibrotic gene products, and significantly reduces the recruitment of CD11b+ macrophages to the lungs. Bronchoalveolar lavage (BAL) cells from RIPF mice show increased Fgr and other senescent cell markers including p16. In human idiopathic pulmonary fibrosis (IPF) and in RIPF, Fgr, and other senescent cell biomarkers are increased. In both mouse and human RIPF, there is an accumulation of Fgr-positive proinflammatory CD11b+ macrophages in the lungs. Thus, elevated levels of Fgr in lung senescent cells upregulate profibrotic gene products, and chemokines that might be responsible for macrophage infiltration into lungs. The detection of Fgr in senescent cells that are obtained from BAL during the development of RIPF may help predict the onset and facilitate the delivery of medical countermeasures.

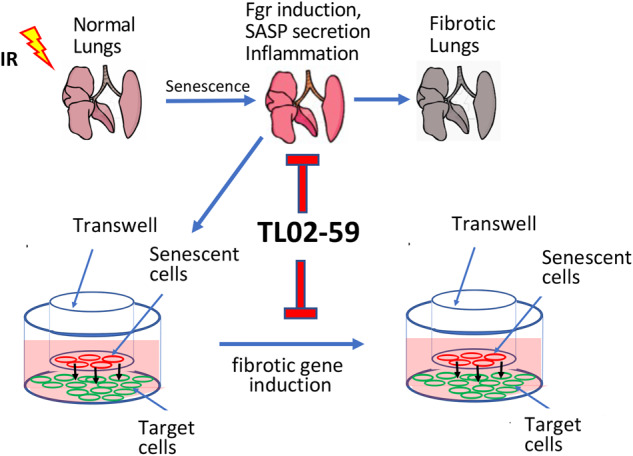

## Introduction

Lung fibrosis can occur for a variety of reasons such as idiopathic pulmonary fibrosis (IPF), radiation-induced pulmonary fibrosis (RIPF), exposure to toxic agents such as silica, and after COVID-19 infection [1–4]. RIPF is incurable, and current antifibrotic therapies have modest effectiveness in slowing the pathogenetic process. A predictive biomarker for the onset of RIPF would aid in the timely delivery of therapeutics to slow the progression of RIPF. Analysis of biomarkers would lead to the discovery of a target to prevent RIPF. Recent evidence suggests that cellular senescence plays a role in both RIPF and IPF [5, 6]. The components of senescence-associated secretory proteins (SASP) have multiple functions. Some components of SASP activate myofibroblasts that deposit collagens, while others recruit inflammatory immunocytes into the lungs [7, 8]. Clearance of senescent cells (SCs) by administration of senolytic drugs has been suggested as a potentially effective therapeutic strategy [9, 10]; however, senescent cells may have beneficial functions in wound repair and tissue regeneration, such that their non-specific elimination may be deleterious [11, 12].

A better strategy to slow the progression of RIPF may be to leave senescent cells intact, but rather eliminate the release of specific deleterious components of SASP. We have shown in purified radiation-induced senescent (RIS) cells from tdTOMp16+ mouse lungs that the expression of a profibrotic tyrosine kinase Fgr is increased. RIS cells induce profibrotic genes in target cells separated by a filter in transwell cultures. Inhibition of Fgr in RIS cells either by shRNA knockdown or by administration of the Fgr inhibitor, TL02-59, abrogates the induction of fibrotic genes in target cells in vitro [13]. In C57BL/6 mice, we have shown that Fgr is upregulated in specific senescent cell phenotypes in RIPF including monocytes, macrophages, and neutrophils [13]. In this report, we tested the hypothesis that inhibition of Fgr prevents RIPF by reducing secretion of proinflammatory chemokines from radiation-induced senescent cells.

## Results

### Senescent cells in mouse RIPF contain Fgr-positive cells that induce profibrotic genes in target cells

**Fig. 1:**
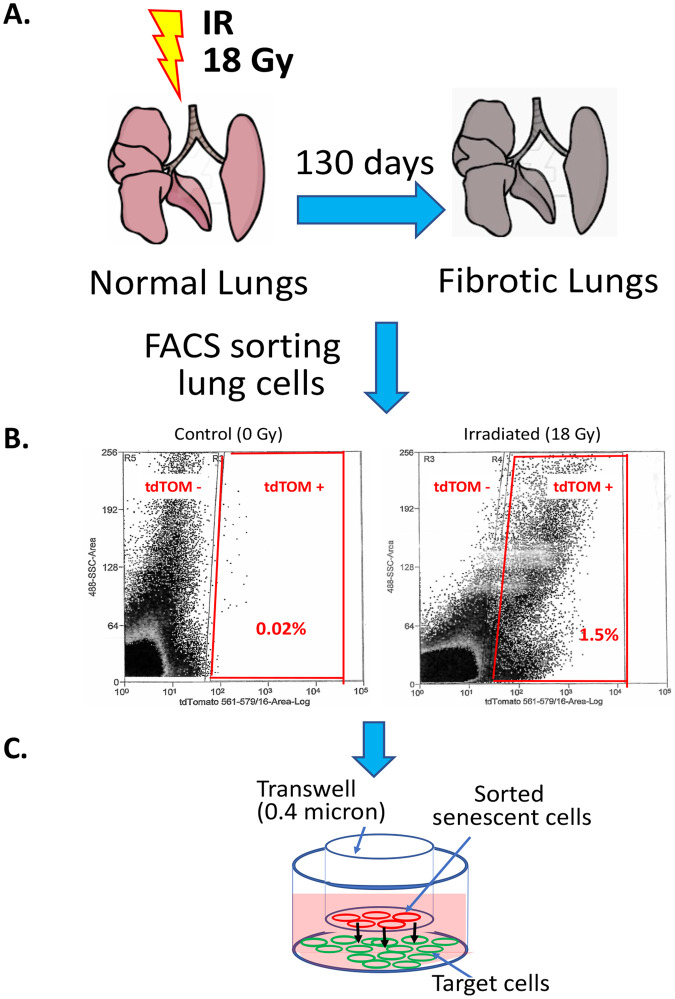
Lung irradiation, senescent cell sorting, and transwell cell culture. **A** Mouse Lungs were irradiated by giving 18 Gy of thoracic irradiation while shielding the rest of the areas of the body. **B** the gating strategy for flow cytometry sorting of tdTOM+ and tdTOM- cells present in the mouse RIPF lungs. **C** non-irradiated, tdTOM- non-senescent, and tdTOM+ senescent cells were cultured in the top well, and the target C57BL/6 fibroblasts were cultured on the bottom well. For qPCR analysis of profibrotic gene expression, the target cells were lysed at day 10 from the bottom well.

To examine the biology of senescent cells in vitro, high doses of radiation and a mixture of senescent and non-senescent cells have been routinely used [14]. We reported that senescent cells can be purified by FACS from irradiated (5 Gy) tdTOMp16+ bone marrow stromal cell line. The tyrosine kinase Fgr-positive senescent cells induced profibrotic gene expression in target cells [13]. To confirm that senescent cells in vivo produced Fgr and induced biomarkers of fibrosis, we FACS purified senescent lung cells from irradiated tdTOMp16+ RIPF mice at 130 days after 18 Gy thoracic irradiation (Fig. 1A).

We isolated senescent (tdTOM+) and non-senescent (tdTOM−) cells from single-cell suspensions of RIPF lungs using flow cytometry (Fig. 1B). In transwells, the top and bottom compartments were separated by a 0.3-micron filter. Non-irradiated lung cells or irradiated lung cells were FACS purified as senescent (tdTOM+) and non-senescent (tdTOM−) cells and were placed in the top chamber of the transwells. In the bottom chamber, we placed C57BL/6 mesenchymal stem cells as target cells and measured fibrosis-associated gene products (Fig. 1C). The Fgr inhibitor TL02-59 (10 nM) was added to the transwell cultures, and at 10 days, we measured the target cells for gene expression using qPCR.

**Fig. 2:**
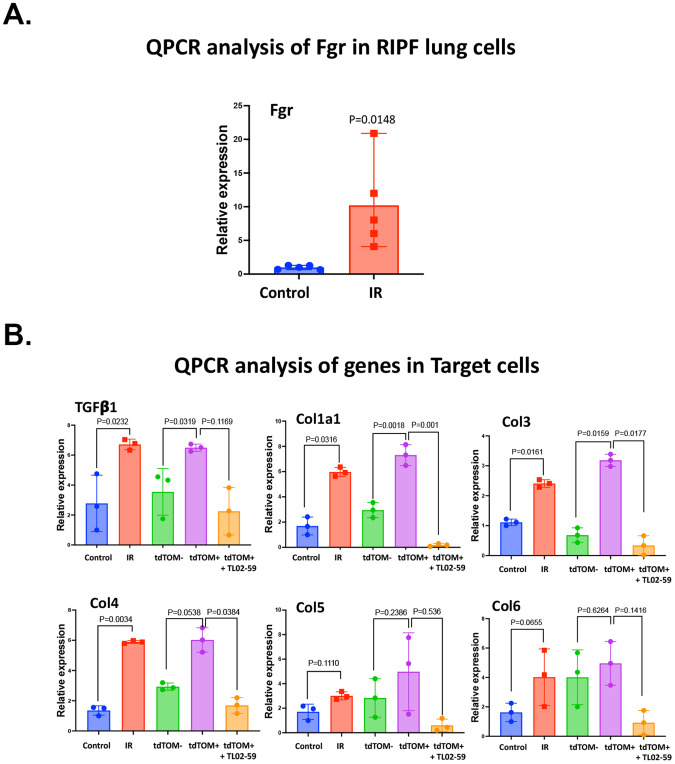
Fgr expression in RIPF lung senescent cells mediates induction of profibrotic gene products in target cells separated in transwell culture. Control and RIPF tdTOMp16+ mouse lungs (0 Gy, and 18 Gy, 130 days) were enzymatically digested to form single-cell suspension, and RIPF lung cells were FACS sorted for irradiated senescent (tdTOM+) and irradiated non-senescent (tdTOM−) cells. **A** Induction of Fgr in control, and thoracic-irradiated lung cells. **B** Upregulation of profibrotic genes (TGF-β, Col1a1, Col3, Col4, Col5, and Col6) in target C57 control cells by thoracic-irradiated lung cells relative to non-irradiated lung cells and FACS purified tdTOMp+ lung senescent cells relative to control, non-senescent tdTOM− cells. The induction of profibrotic genes is abrogated by TL02-59 treatment (10 nM) for 10 days. (*n* = 3, *P* values were calculated by non-parametric *t* test).

Irradiated lung cells showed significant upregulation of Fgr (Fig. 2A). Induction of profibrotic genes (Tgf-beta, Col1a1, 3, 4, 5, and 6) in target cells was detected with irradiated non-purified or purified senescent cells in the top well (Fig. 2B). The Fgr inhibitor TL02-59 significantly reduced the levels of profibrotic gene products in target cells. Thus, purified senescent cells from the lungs of thoracic-irradiated p16tdTOM+ mice induced profibrotic genes in target cells. The induction was abrogated by the Fgr inhibitor TL02-59. These data confirm and extend prior results with irradiation-induced senescent bone marrow stromal cells that were obtained from tdTOMp16+ mice [13].

### Inhibition of Fgr reduces expression of fibrotic genes in thoracic-irradiated mouse lungs

**Fig. 3:**
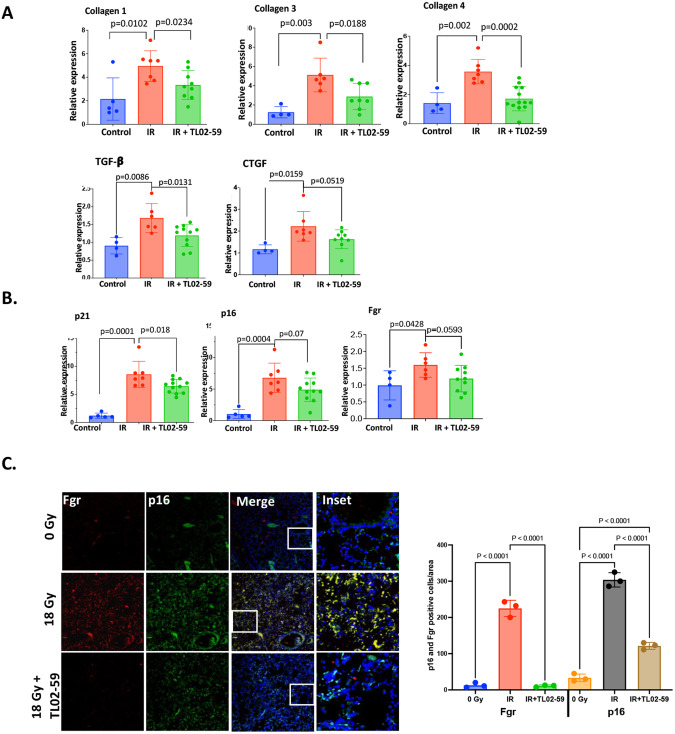
Fgr inhibitor TL02-59 reduces levels of profibrotic gene products and p16 in thoracic-irradiated mouse lungs. Thoracic-irradiated (18 Gy) C57BL/6 mice were treated with either vehicle or Fgr inhibitor TL02-59 (10 mg/kg) by oral gavage at day 50 for 4 weeks (3 times a week, alternative days). At Day 130 after irradiation, the mice were sacrificed and one lobe of the lungs were collected for qPCR assessment. **A** Expression of fibrotic genes (Collagen 1, Collagen 3, Collagen 4, TGF-β, and CTGF) were assessed by qPCR. **B** Expression of Fgr and senescent biomarkers p16, p21 were assessed by qPCR. **C** Immunofluorescence staining of the lungs showing Fgr (Green), p16 (Red), and colocalized (yellow) lung cells from control, thoracic-irradiated, and TL02-59 treated. Quantification of Fgr and p16-positive cells are shown on the right. (*n* = 4–10 mice, *P* values were obtained by non-parametric *t* test, IF quantification was done using cells/equivalent areas (*n* = 3), *p* values were calculated by ANOVA, Turkey’s multiple comparison test).

During the first 50 days after thoracic irradiation, there is no apparent lung fibrosis [13, 15]. However, beginning at day 50 there is a progressive increase in tyrosine kinase Fgr and other biomarkers of senescent cells including p16 and p21 [13]. Thoracic-irradiated mice received Fgr inhibitor TL02-59 (10 mg/kg) three times a week for 4 weeks beginning at day 50. qPCR was used to assess levels of fibrotic gene transcripts in mouse lungs for control (0 Gy), and thoracic-irradiated mice (18 Gy, day 130). There was upregulation of Tgf-β, Ctgf, Collagen 1a1, Collagen 3, and Collagen 4 in RIPF lungs compared to the control non-irradiated lungs (Fig. 3). The expression of these genes were significantly reduced in the lungs of mice that had been treated with TL02-59 (Fig. 3A). The levels of Fgr and other senescent cell biomarkers such as p16 and p21 were significantly increased in irradiated mouse lungs relative to control non-irradiated lungs. There was a significant downregulation of p21, p16, and Fgr in TL02-59 treated mouse lungs (Fig. 3B). Immunofluorescence staining showed significant induction of both Fgr and p16 in histopathologic sections of thoracic-irradiated mouse lungs. The induction was reduced by TL02-59 treatment (Fig. 3C). These data establish that inhibition of Fgr in thoracic-irradiated mice reduces the levels of expression of pre-fibrotic gene products, and other senescent cell biomarkers.

### Fgr inhibitor TL02-59 treatment reduces mouse RIPF

**Fig. 4:**
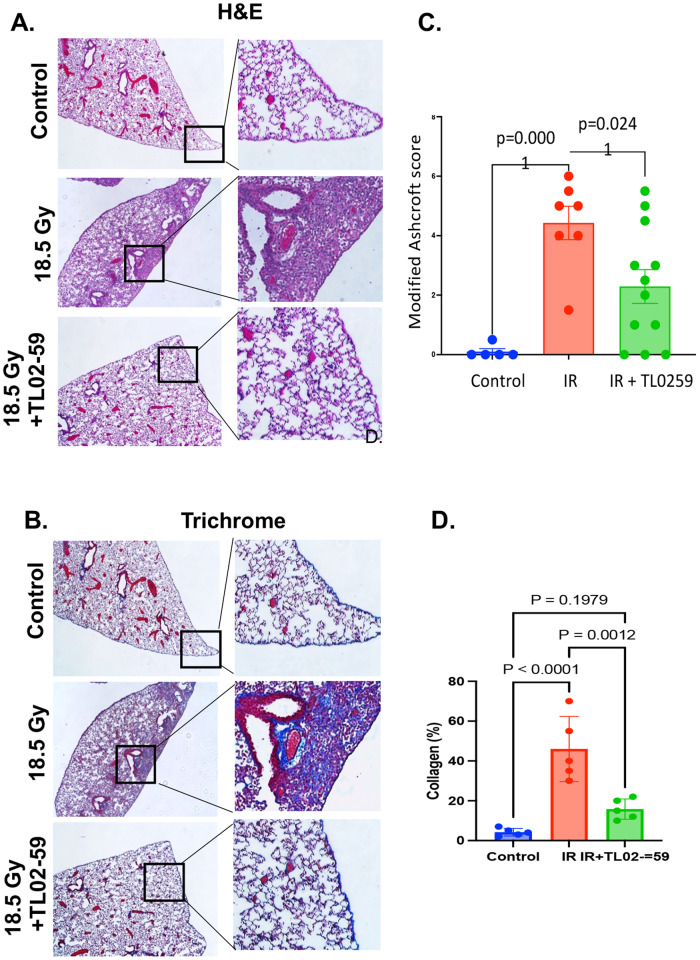
Fgr inhibitor TL02-59 reduces mouse RIPF. Thoracic-irradiated (18 Gy) C57BL/6 mice were treated with either vehicle or Fgr inhibitor TL02-59 (10 mg/kg) by oral gavage at day 50 for 4 weeks (3 times a week, alternative days) and at Day 130 mice were sacrificed and lungs were collected in PFA for histological analysis. **A** Representative images with magnified insets of mouse lungs stained with H&E. **B** Representative images with magnified insets of mouse lungs stained with Masson’s trichrome staining. **C** Grading of fibrosis from H&E-stained lungs using modified Ashcroft score^1^ was performed in a blinded fashion. Grading shows significant induction in lung fibrosis in thoracic-irradiated lungs relative to non-irradiated lungs and fibrosis was significantly reduced in TL02-59 treated lungs. **D** Blue Masson’s trichrome staining was quantified by ImageJ software (*n* = 5–12; *p* values were calculated by ANOVA, Turkey’s multiple comparison test).

Next, we evaluated whether TL02-59 treatment given at the time of detectable increase in Fgr (day 50 [13]) after thoracic irradiation (18 Gy) for 4 weeks reduced RIPF. TL02-59 is orally bioavailable, and 10 mg/kg dose was effective in mice to irradicate leukemic cells entirely [16]. Hence, we chose to gavage TL02-50 at a dose of 10 mg/kg. At day 130 after irradiation, we sacrificed control (0 Gy), thoracic-irradiated (18 Gy), and thoracic-irradiated and TL02-59 (10 mg/kg) treated mice. We quantitated RIPF in sections of lungs stained with hematoxylin and eosin (H&E), and Masson’s trichrome under light microscope (Fig. 4A, B). Using the modified Ashcroft scale [17, 18] and blinded scoring, the grades of fibrosis showed a significant increase in fibrosis after 18 Gy thoracic irradiation by day 130 mice compared to control non-irradiated mice. There was a statistically significant reduction in RIPF in thoracic-irradiated mice that were treated with TL02-59 (Fig. 4C). Analysis of the trichrome-stained lung sections for collagen-positive areas (Fig. 4D) using ImageJ software revealed that blue collagen-positive areas were significantly induced in the lungs of thoracic-irradiated mice and reduced by TL02-59. Thus, the Fgr inhibitor TL02-59 reduced RIPF in thoracic-irradiated mice.

### Fgr inhibitor TL02-59 reduces RIS lung cell release of chemokines that are part of SASP proteins

**Fig. 5:**
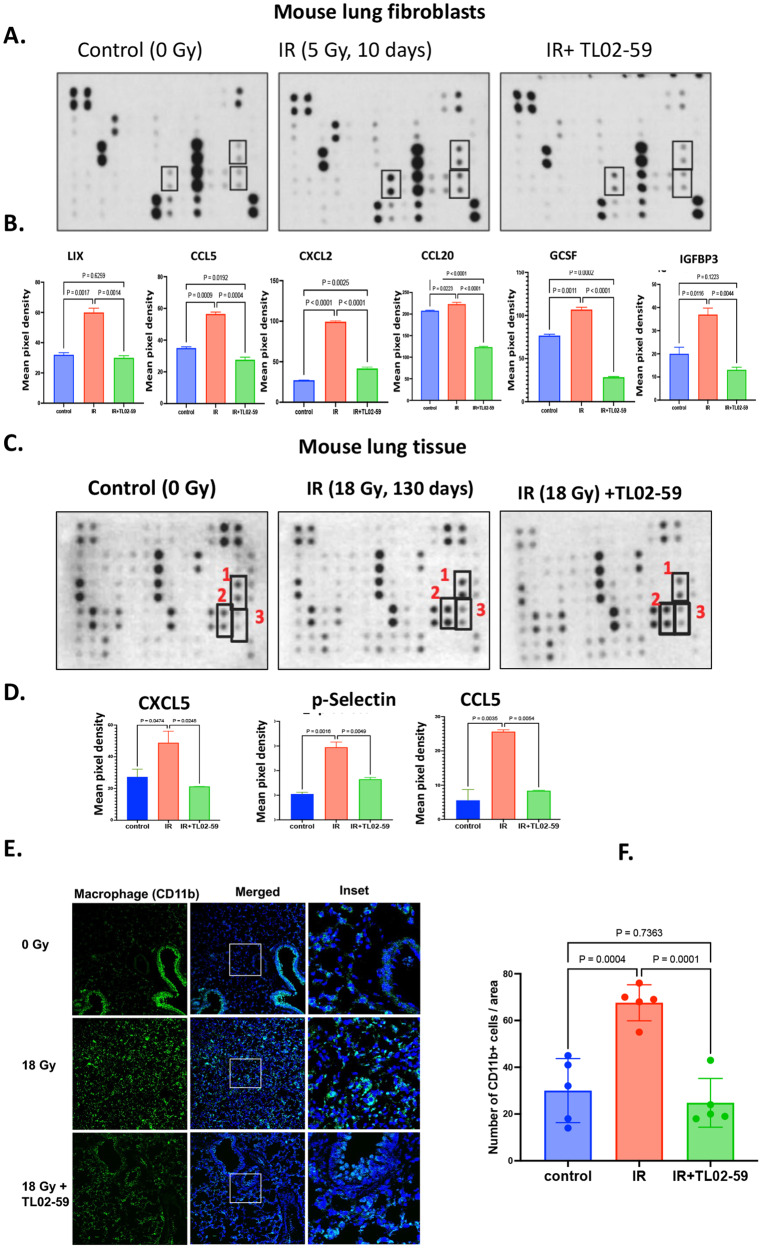
Fgr inhibitor TL02-59 reduces secretion of chemokines in the senescence-associated secretory proteins from RIS lung cells in vitro. **A** Isolated primary lung fibroblasts from C57BL/6 mice were irradiated (5 Gy) in culture. 10 days after irradiation the cells were replated and cultured with either vehicle or TL02-59 (10 nM) for 72 h. Media was collected and a protein array for 62 secreted chemokines was performed. **B** Relative amounts of chemokines secreted in the media were quantified using manufacturer’s quantification algorithm. LIX, CXCL5, CCL5, CXCL2, CCL20, G-CSF, and IGFBP-3 were present in increased amounts in the media of irradiated cells and significantly reduced with 72 h or TL02-59 treatment. **C** Thoracic-irradiated (18 Gy) C57BL/6 mice were treated with either vehicle or Fgr inhibitor TL02-59 (10 mg/kg) by oral gavage at day 50 for 4 weeks (3 times a week, alternative days) and at day 130 mice were sacrificed and lung tissues were collected for cytokine array analysis. **D** Relative amounts of chemokines present in the lung lysate were quantified (CXCL5, CCL5, and p-selectin were unregulated in RIPF lungs and downregulated in IR+ thoracic-irradiated lungs. **E** CD11b Immunofluorescence showing increased CD11b+ alveolar macrophage in the RIPF lungs and decreased in the IR + TL02-59 lungs. **F** Quantification of CD11b positive cells is shown on the right. (*n* = 3, *p* values were calculated by ANOVA, Turkey’s multiple comparison test).

We have analyzed differentially expressed genes (DEGs) using RNA-seq and found genes that are related to chemotaxis were significantly upregulated (Fig. S1A) in senescent p16tdTOM+ bone marrow stromal cells relative to non-senescent p16tdTOM- cells. Gene ontology (GO) analysis showed that chemotaxis-related DEGs were significantly enriched in radiation-induced senescent cells compared to both radiation-induced non-senescent cells (Fig. S1B, C) and non-irradiated cells. We next tested whether inhibition of Fgr would reduce chemokine release from senescent cells using purified sorted senescent cells from irradiated tdTOMp16+ bone marrow stromal cells. We tested the effect of TL02-59 treatment in vitro for 72 h. We measured the chemokine levels in the culture media using chemokine array. The chemokines CXCL1, CXCL5, and CCL-2 were upregulated in the media of senescent cells and were significantly reduced in Fgr inhibitor TL02-59 treated cells (Fig. S2). We demonstrated that isolated mouse primary lung fibroblasts exhibited senescence 10 days after 5 Gy (Fig. S3). We confirmed in Fig S3C that the cells were of fibroblast origin due to the expression of actin stress fiber (Fig S3C). We then irradiated mouse primary fibroblasts to 5 Gy for 10 days and cultured the cells for 72 h after replating with and without TL02-59 treatment (10 nM). We quantitated the chemokine levels in the media using chemokine array [19]. We found that chemokines LIX, CXCL5, CCL5, CXCL2, CCL20, G-CSF, and IGFBP-3 were present in increased amounts in the media of radiation-induced senescent cells compared to that of non-irradiated cells. TL02-59 treatment significantly reduced the levels of these chemokines (Fig. 5A, B).

We next performed chemokine array analysis on mouse lungs from control (0 Gy) mice, irradiated mice developing RIPF (18 Gy, day 130), and thoracic-irradiated mice that were Fgr inhibitor-treated (10 mg/kg, at day 50, three times a week for 4 weeks). We detected upregulation of chemokines CXCL5, CCL5, and p-Selectin in RIPF lungs compared to the control non-irradiated lungs, and the upregulation of these proteins was reduced in the lungs of mice treated with TL02-59 (Fig. 5C, D).

### Fgr inhibitor TL02-59 reduces levels of infiltrating inflammatory macrophages in thoracic-irradiated mouse lungs

It is established that CD11b+ macrophages and neutrophils increase inflammation in the lungs during lung injury [20]. We next quantitated the numbers of CD11b+ cells in RIPF lungs relative to control non-irradiated and TL02-59 treated lungs of mice. Immunofluorescence staining showed an increase in CD11b+ cells in the RIPF lungs compared to the control lungs. Compared to lungs after TL02-59 treatment, infiltration of CD11b+ leukocytes was significantly reduced (Fig. 5E, F) The data indicate that Fgr inhibitor TL02-59 treatment led to a decrease in the secretion of chemokines which may have resulted in reduced CD11b+ inflammatory leukocyte infiltration.

### Fgr inhibitor TL02-59 treatment reduces Fgr and p16 induction in BAL cells in thoracic-irradiated mouse lungs

**Fig. 6:**
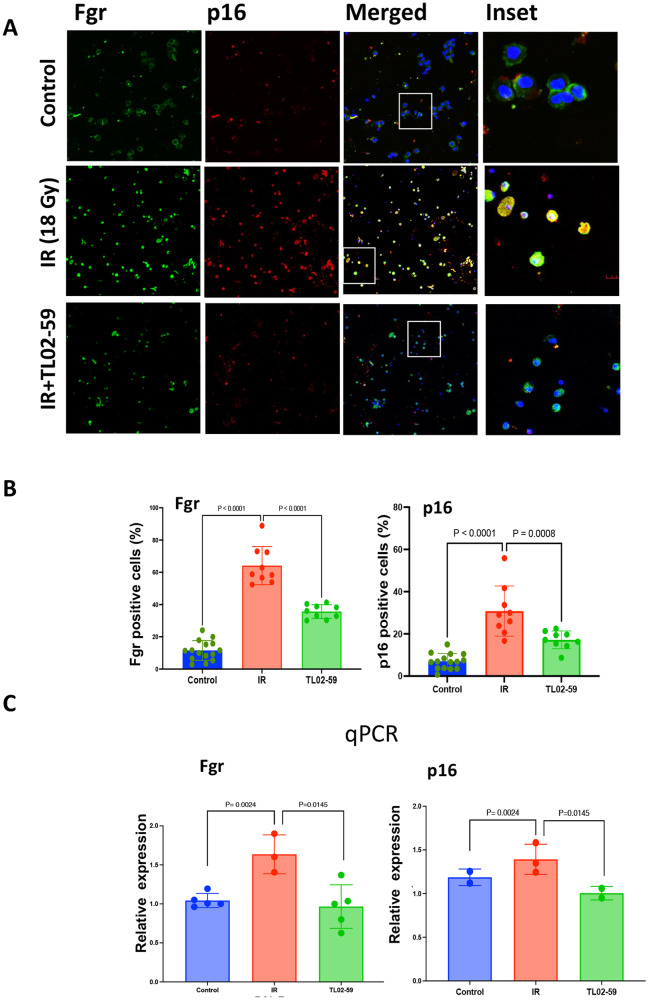
Detection of Fgr and p16 in bronchoalveolar lavage (BAL) cells from mice that are developing RIPF. BAL samples were collected from age-matched (1) non-irradiated control (C57BL/6), (2) thoracic-irradiated (IR, 18 Gy), (3) IR + Fgr inhibitor TL02-59 (10 mg/kg at day 30 after IR, 3 days a week for 4 weeks). BAL samples were collected at day 130 after radiation when lung fibrosis is evident. The BAL cells were either stained for Fgr and p16 or used to isolate RNA and perform qPCR. Images are representative of *n* = 3 for each group. **A** Immunoflurescence, **B** quantification of Fgr and p16-positive cells. **C** qPCR for p16 and Fgr was performed from BAL cells. (One-way ANOVA, Turkey’s multiple comparison test was used to obtain the *p* values).

Bronchoalveolar lavage (BAL) consists of cells and fluid that have previously been reported to be valuable in diagnosing the onset of inflammation during the pathogenesis of lung fibrosis [21]. We measured the expression of Fgr and senescent marker p16 in the BAL cells from non-irradiated, thoracic-irradiated (18 Gy), and TL02-59 treated (10 mg/kg three times a week for 4 weeks, beginning at day 50) thoracic-irradiated mice at their experimental end point at day 130. We performed immunofluorescence staining of BAL cells that were fixed on slides by cytospin. There was a significant increase in Fgr and p16 positive cells in RIPF mice, compared to non-irradiated mice, and were significantly reduced in the BAL of thoracic-irradiated TL02-59-treated mice (Fig. 6A, B). qPCR analysis of BAL cells showed an increase in Fgr and p16 in cells from irradiated mice compared to control mice and the levels were significantly reduced in TL02-59 treated mice (Fig. 6C). Thus, senescent cell markers p16 and Fgr are detected in BAL cells harvested from thoracic-irradiated RIPF mice and levels are reduced by the treatment with TL02-59.

### The small molecule radiation mitigator, MMS350 reduces lung senescence and subsequently inhibits profibrotic gene expression in thoracic-irradiated mice

**Fig. 7:**
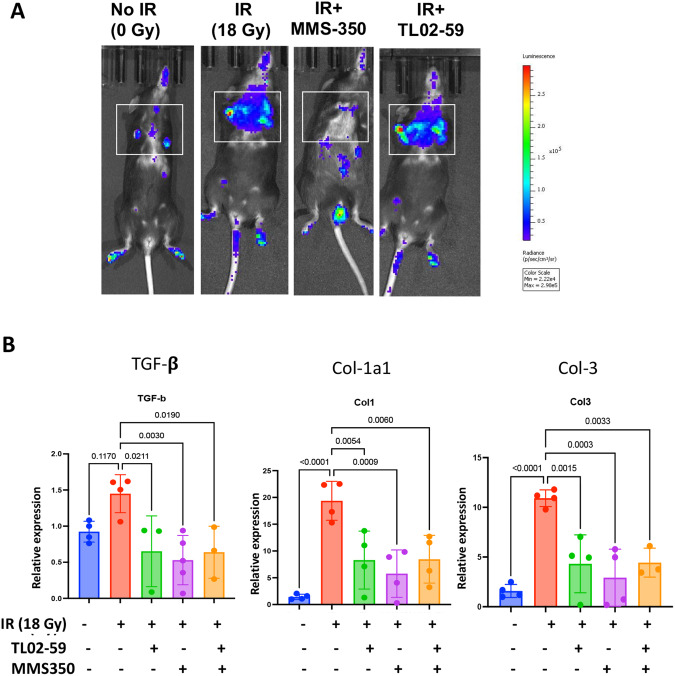
Administration of MMS350 reduce numbers of lung senescent cells and profibrotic gene product levels in thoracic-irradiated mice. **A** Expression of luciferase gene in control (No IR), irradiated (IR), IR + MMS350, and IR + TL02-59 in thoracic-irradiated p16-LUC+ mice at day 120. **B** Expression of TGF-β, Collagen 1a1, and Collagen 3 in non-irradiated control (C57BL/6), thoracic-irradiated (IR, 18 Gy), (3) IR + Fgr inhibitor TL02-59 (10 mg/kg at day 30 after IR, 3 days a week for 4 weeks), (4) IR + MMS350 (100ug/ml in drinking water) and (5) IR + TL02-59 + MMS350 treated mice as measured by qPCR. *p* values were obtained by one-way ANOVA. (*n* = 4–5).

We evaluated whether a drug that reduces the number of senescent cells in irradiated mouse lung (MMS350) [22, 23] blocked Fgr. Since shRNA silencing of Fgr did not block irradiation-induced senescence, we tested whether another method to remove senescent cells reduced Fgr (13). We irradiated transgenic p16-LUC+ mice where the luciferase gene is under the control of one allele of p16 gene promoter [22]. We compared the effect of TL02-59 with MMS350 in thoracic-irradiated mice. We used MMS350 given in the drinking water (400 µM) [23] continuously beginning one week before irradiation. We followed mice for the induction of luciferase gene in p16-LUC+ mice using the IVIS imaging system during the course of RIPF pathogenesis in control (No IR), irradiated (IR), IR + MMS350, and IR + TL02-59 in thoracic-irradiated p16-LUC+ mice weekly. We observed that TL02-59 treatment did not have any effect on luciferase expression although MMS350-treated mice showed a significant reduction of luciferase expression in thoracic-irradiated p16-LUC+ mice (Fig. 7A). Both MMS350 and TL02-59 reduced the expression of markers of fibrosis (TGF-β, Collagen 1a1 and Collagen 3) compared to control thoracic-irradiated (IR, 18 Gy) mice (Fig. 7B). The data indicate that reduction of the numbers of senescent cells by treatment with MMS350 prevents RIPF. However, it was not necessary to remove the senescent cells to prevent RIPF by using TL02-59.

### Fgr and other senescent cell biomarkers are increased in the lungs from patients with IPF and RIPF

**Fig. 8:**
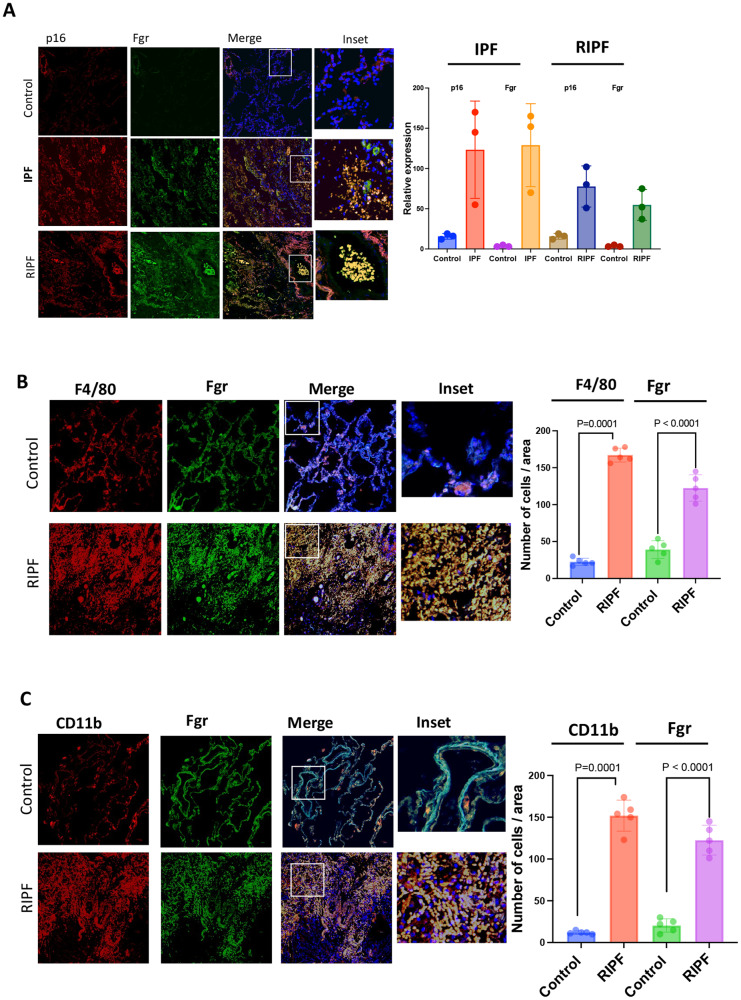
Senescent cell numbers and Fgr-positive cells are increased in IPF and RIPF patient lung histopathologic sections. **A** Surgically resected human lungs and age-matched control lungs were stained for Fgr and p16. Third panel shows merged images of Fgr and p16 with blue nuclear stain DAPI (×20). Far right column shows enlarged areas (boxes). Images are representative of samples from three patients in each lung pathology. P16 and Fgr-positive cells were quantified from both IPF and RIPF sections. **B** Human RIPF sections were stained with Fgr and co-stained with F4/80 for monocytes, macrophages, and dendritic cells. **C** Human RIPF sections were stained with Fgr and co-stained with CD11b for inflammatory alveolar macrophages. Quantification of Fgr and p16-positive cells are shown on the right. (*n* = 3, *p* values were calculated by ANOVA Turkey’s multiple comparison test).

We next analyzed the levels of Fgr expression in the published human lung disease cell atlas database and observed that Fgr is also upregulated in interstitial lung disease (ILD) (Fig. S4A), and idiopathic pulmonary fibrosis (IPF) (Figs. S4B, 8), relative to control lungs. Fgr expression was primarily restricted in monocytes, macrophages, and dendritic cells as we have observed in the single-cell RNA-seq analysis of mouse RIPF [13]. To confirm that upregulation of Fgr similarly occurs in human RIPF, we obtained fibrotic lungs from patients that were treated with radiation for lung cancer but returned with severe lung fibrosis. Surgically resected lungs of radiotherapy patients and patients with IPF and age-matched control lungs were immunostained for Fgr, and the senescent cell marker p16 (Fig. 8A). Severe lung fibrosis in patients with RIPF was confirmed by trichrome staining (Fig. S4C). In histopathologic sections of both human RIPF and IPF lungs, the levels of Fgr were significantly higher in cells that were also positive for senescent marker p16. CD11b^high^ alveolar macrophages are known to accumulate in patients with inflammatory lungs [20]. F4/80 is highly expressed in resident tissue macrophages, and Fgr has been shown to express in myeloid origin cells [16]. We immunostained human RIPF lungs with Fgr, F4/80, and CD11b and found that CD11b and F4/80 positive cells were abundant in human RIPF lungs compared to age-matched control lungs (Fig. 8B, C). Fgr-positive cells were also positive for CD11b and F4/80. Therefore, our data suggest that in human RIPF lungs, Fgr is present in cells that are positive for the senescent marker p16 and other markers for inflammatory macrophages.

## Discussion

RIPF and IPF are irreversible conditions. The available antifibrotic drugs are currently directed to slow the progression of the disease. Multiple tyrosine kinases (both receptor and non-receptor tyrosine kinases) have been implicated in fibrosis progression [24]. Clinical studies targeting these tyrosine kinases have reported early modest effects [25]. The FDA-approved IPF drug Nintedanib is an inhibitor of multiple receptor tyrosine kinases including the platelet-derived growth factor receptor (PDGFR), the vascular endothelial growth factor receptor (VEGFR), and the fibroblast growth factor receptor (FGFR) [26, 27]. In clinical trials, 95% of the patients discontinued Nintedanib treatment due to severe side effects; perhaps, attributable to off-target effects [27].

Cellular senescence has been linked to IPF and RIPF [5–8]. We have previously demonstrated that in mouse RIPF, senescence precedes lung fibrosis. Since the benefits of senescence have been made abundantly clear in recent studies [11, 28], our primary goal was to identify a molecular regulator that controls the inflammatory nature of senescent cells. Using pure RIS cells, and mouse RIPF lungs, we previously identified a non-receptor tyrosine kinase Fgr, which is induced in senescent cells [13]. Here we sorted pure senescent cells from thoracic-irradiated tdTOMp16+ mice at their experimental endpoint. These cells, when co-cultured with target lung fibroblasts in a non-contact transwell system, induce profibrotic genes in target cells which is abrogated by the small molecule TL02-59 which is a specific inhibitor or Fgr (Fig. 2). Pharmacokinetic studies demonstrated that TL02-59 is orally bioavailable and specifically inhibits Fgr in nanomolar concentration [16]. Here we show that an early intervention, targeting Fgr with TL02-59, prevents pulmonary fibrosis in thoracic-irradiated mice and abrogates the induction of fibrotic biomarkers of fibrosis in the lung tissue.

In response to ionizing irradiation, bone marrow leukocyte recruitment into the lungs is associated with lung damage [29]. It has been shown that Fgr and tyrosine kinase Hck double knockout mice are unable to recruit neutrophils and monocytes to the lungs after bacterial lipopolysaccharide (LPS) induced injury. Moreover, this defect is attributed to the reduction in chemokine secretion from the lungs and not due to any intrinsic defect of cell locomotion [30]. We have analyzed differentially expressed genes (DEGs) using RNA-seq and found a sizable list of genes related to chemotaxis were significantly upregulated in senescent p16tdTOM+ bone marrow stromal cells relative to non-senescent p16tdTOM- cells. Gene ontology (GO) analysis showed that chemotaxis-related DEGs were significantly enriched in radiation-induced senescent cells compared to both radiation-induced non-senescent cells and non-irradiated cells. Chemokines CXCL5 and CCL5 are known to play a role in leukocyte chemotaxis during inflammation of the lungs [31, 32] and CXCL5 has been associated with idiopathic pulmonary fibrosis. Moreover, elevated levels of CXCL5 were detected in the BAL fluid of IPF patients [28, 31]. Fgr was shown to be a driver of pathogenic neutrophils. Depletion or inhibition of the Fgr kinase by TL02-59 has protected the lungs from neutrophil-mediated inflammatory injury [33]. We evaluated the possible link between Fgr and the secretion of inflammatory factors by senescent cells in irradiated lung fibroblasts and in thoracic-irradiated lung tissues. The chemokines (CXCL5, CCL5, CXCL2, CCL20, and G-CSF) were significantly upregulated by irradiation-induced senescent cells and were downregulated by TL02-59. All of these chemokines have been associated with inflammatory disease in the lungs. CXCL5 has been associated with several inflammatory and fibrotic diseases including IPF (elevated levels were detected in the BAL fluid of IPF patients) [26, 34]. CCL5 is known to be a profibrotic SASP while increased expression of CXCL2 is strongly associated with radiotherapy-induced lung fibrosis. CCL20 is involved in the pathogenesis of ILD and COPD, and Granulocyte-CSF links destructive inflammation and comorbidities in obstructive lung disease [35, 36]. In line with these results, our immunofluorescence staining of human and mouse RIPF lungs showed increased Fgr and CD11b positive macrophages. However, to establish that the recruitment of inflammatory immunocytes to the lungs takes place following the release of specific chemokines from lung senescent cells future studies are required. In TL02-59 treated mouse lungs, the number of these immunocytes was significantly reduced. Fgr is expressed primarily in monocytes, neutrophils, macrophages (13), and lung fibroblasts. We found that Fgr is increased in RIPF-associated senescent cells.

Fgr is a member of the Src family of non-receptor tyrosine kinases and has been described to be overexpressed in many human cancers [16]. Fgr is upregulated in human IPF, interstitial lung disease (ILD), and Chronic Obstructive Pulmonary Disease (COPD) relative to control human lungs [23, 37]. In other studies, depletion, or inhibition of Fgr kinase using TL02-59 protects neutrophil-mediated inflammatory injury, and Fgr knockout (Fgr-/-) mice were reported to have significantly reduced ischemia injury-induced myocardial fibrosis [34]. Moreover, Fgr kinase is involved in macrophage activation in proinflammatory adipose tissue, diet-induced obesity, insulin resistance, and liver steatosis [38].

We postulated that Fgr is involved in fibrosis downstream of senescence. We hypothesized that blocking of Fgr would not alter senescence but blocking of senescence would remove the induction of Fgr. We confirmed this hypothesis in thoracic-irradiated p16-LUC+ mice. We used TL02-59 and the small molecule drug, MMS350, which also prevents RIS in vitro and in vivo, and confirmed that senescence was independent of Fgr. The inhibition of Fgr did not block the induction of senescence. However, the removal of senescent cells by MMS350 did remove the effect of Fgr by reducing the expression of fibrotic genes (Fig. 7B). We documented a slight decrease in biomarkers of senescence in TL02-59 samples. One possibility is that by reducing the secretion of proinflammatory SASP, TL02-59 may have reduced a paracrine effect of senescence which induces senescence in other cells. The mechanism by which Fgr is induced in senescent cells is unknown. One possibility is that transcription factors that are upregulated during radiation-induced DNA damage such as p53 or AP1, may bind to the putative binding sites on Fgr promoter and induce Fgr. Studies to test this hypothesis are in progress. Moreover, It is imperative that to predict pulmonary fibrosis or to treat human RIPF or IPF more preclinical data and validation of the involvement of Fgr in other models of IPF is warranted.

Slowing the progression of RIPF in humans will require early detection of its onset and timely therapeutic intervention. We have shown that the cells that are present in BAL fluid of RIPF mice express high levels of Fgr and p16 than that of control mice, and in TL02-59 treated thoracic-irradiated mice the expression of Fgr and p16 was reduced in the BAL cells reflects our observation in the lung tissue. Monitoring BAL cells and fluids for Fgr and senescence biomarkers may predict the severity or time of onset of RIPF allowing thoughtful initiation of therapy with an anti-fibrotic agent.

## Methods

### Mice

The mouse strain tdTOMp16+ was obtained from Dr. B. O. Diekman, University of North Carolina at Chapel Hill [39], p16+/LUC mice on the C57BL/6 J background were obtained from Dr. Norman Sharpless [40]. C57BL/6 mice were purchased from Taconic Biosciences (Germantown, NY). All animal protocols used were approved by the University of Pittsburgh’s Institutional Animal Care and Use Committee (IACUC). Veterinary care was provided by the University of Pittsburgh Division of Laboratory Animal Resources (DLAR).

### Monitoring of p16^+/LUC^ mice for senescence in lungs

Following thoracic irradiation of 18 Gy, p16^+/LUC^ mice were serially imaged using the Lumina XR System (IVIS) imaging system (Perkin Elmer, Waltham, MA) for luciferase+ areas in the lungs, at 10 min after injection of luciferin. Serial images demonstrated the appearance of senescent areas throughout the lungs.

### Irradiation

Thoracic irradiation was carried out according to published methods using a Varian TrueBeam linear accelerator [22]. The mice were irradiated using an irradiation field of 2 cm × 40 cm. The mice were placed in the irradiation field so that only the thoracic cavity was irradiated with the remainder of the mouse shieled from the irradiation. The mice were irradiated using a source-to-surface distance (SSD) of 100 cm using 6 MV photons at an irradiation dose of 600 monitor units (MU) per minute. For the mouse thoracic irradiation experiment, a total of 80 C57BL/6 mice were randomly divided into control and experimental groups (irradiated, irradiated, and drug-treated) and sacrificed when moribund or at day 130. The schema for the irradiation experiments is shown in Fig. 1.

### Drug delivery

TL02-59 was purchased from Med Chem Express in a powder form (Cat. No.: HY-112852). MMS350 [37] was obtained from Dr. Peter Wipf of the Department of Chemistry of the University of Pittsburgh. Phalloidin-FITC for actin staining was purchased from Tocris (Cat. No. 5782, Minneapolis, MN). TL02-59 was gavaged 3 times a week for 4 weeks at 10 mg/kg beginning on day 50. MMS350 was administered in drinking water at 400 µM beginning one week before irradiation until the mice were sacrificed.

### Human lung tissue

Paraffin-embedded human lung tissues of normal, IPF, and RIPF patients were obtained from the University of Pittsburgh’s Biospecimen Core as approved by the University of Pittsburgh’s Internal Review Board (IRB).

### Antibodies for immunohistochemistry

Antibody to Fgr (sc-74542 and for humans, sc-166079), and F4/80 (sc-377009) for staining mouse and human lungs were purchased from Santa Cruz Biotechnology, Inc. (Dallas TX, for mice,). A second F4/80 antibody (70076 T) was purchased from Cell Signaling Technology (Danvers, MA). p16 antibody (10883-1-AP) was purchased from Proteintech (Rosemont, IL). CD11b (557960) antibody was purchased from BD Biosciences (Franklin Lakes, NJ).

### Lung histology

Mice were sacrificed at day 130 post-irradiation. The lungs and trachea exposed with the left lobe of the lungs tied off with suture thread and the other lobes of the lung inflated by injecting 10% paraformaldehyde intratracheally. The left lobe was cut off and immediately frozen in liquid nitrogen for mRNA and protein analysis. The remainder of the lungs were fixed by placing them in 10% paraformaldehyde, parafilm embedded, and sectioned for histochemical analysis. Lung tissue was also removed at select time points for histology according to published methods [23, 41].

### Isolation of mouse primary lung fibroblasts

Mouse fibroblast cells were isolated, characterized, and maintained following the protocol described by Edelman and Redente [42]. Lungs from 12-week-old mouse were minced, digested using collagenase A and maintained in a Fibroblast culture medium: 500 ml DMEM supplemented with 200 mM L-Glutamine (final concentration), 10 mL Pen-Strep (10,000 U/mL penicillin, 10,000 U/mL streptomycin). All experiments were performed using cells that were in culture between 4 to 10 passages. To confirm that the isolated lung fibroblasts as grown in culture were of lung origin expressing F-actin stress fibers we stained the cells grown in chambered slides with phalloidin (green) and DAPI-stained nuclei (blue).

### Transwell cocultures

A Transwell system (0.4-μm pore size, polyester membrane; Corning, Kennebunk, ME) was used [43]. C57BL/6 mouse primary lung fibroblasts were cultured on the bottom chamber surface of a 9-cm^2^ culture dishes (Fig. 1). Above the adherent layer in the transwell, non-irradiated, irradiated non-sorted, irradiated, and sorted senescent tdTOM +, or irradiated non-senescent lung cells from p16tdTOM+ mice were cultured. For the TL02-59 experiment, the drug (10 nM) was added to the media at the time the cells were plated on the transwell. Briefly, tdTOMp16+ mice were thoracic-irradiated (18 Gy) and at day 130, cells were FACS sorted for tdTOM+ and tdTOM- cells, and 3 × 10^5^ cells were added on the top wells in each case. Aliquots of lung cells were kept for the confirmation of Fgr induction by qPCR. Co-culture was maintained for 10 days. The target cells from the bottom chamber were lysed, and total RNA was isolated using TRIzol (Invitrogen, Life Technologies, ThermoFisher Scientific, Waltham, MA) reagent following the manufacturer’s protocol.

### RNA isolation and cDNA synthesis

Total RNA was isolated from respective cell lines (tdTOMp16 + lung cells, C57BL/6 lung primary fibroblasts) according to the protocol supplied with TRIZOLl Reagent (Invitrogen, Life Technologies, ThermoFisher Scientific, Waltham, MA). The concentrations of the RNA samples were determined using a spectrophotometer and cDNAs were made from RNA (2 μg) using a high-capacity RNA-to-cDNA™ Kit (ThermoFisher Scientific, Waltham, MA) following the manufacturer’s instructions.

### Real-time PCR

Quantitative reverse transcription-PCR (qRT-PCR) was performed using Biorad CFX-connect Real-Time System instrument (Biorad, Hercules, CA) and commercially available target probes and Master mix (all from Applied Biosystems, ThermoFisher, Waltham, MA). Detection of mouse Fgr, p16 (CDKN2A), p21 (CDKN1A), p19, Collagens (1 and 3), TGF-beta, α-smooth muscle actin (Acta 2), CTGF, and GAPDH were achieved using specific Taqman Gene Expression Assays (Mm00438951_m1, Mm00494449_m1, Mm04205640_g11, Mm01191861_m1, Mm01192933_g1Mm01257348_m1, Mm00600638_m1, Mm00725412_s1, Mm00802305_g1, Mm99999915_g1, respectively). Real-time reactions were run using the following cycling parameters: 95 °C for 12 min, followed by 40 cycles of 95 °C for 15 s and 60 °C for 1 min. Differential gene expression was calculated by the ΔΔCT calculation [44].

### Generation of single-cell suspensions from whole mouse lung

The lungs of mice were first inflated with 1 ml of sterile PBS and allowed to collapse, and then the lungs were inflated with the enzyme mix containing dispase (50 caseinolytic units/ml), collagenase (2 mg/ml), elastase (1 mg/ml), and DNase (30 μg/ml). The lungs were removed and immediately minced into small pieces (~1 mm^2^). The tissue was transferred into 10 ml enzyme mix for enzymatic digestion for 30 min at 37 °C. Enzyme activity was inhibited by adding 5 ml of phosphate-buffered saline (PBS) supplemented with 10% fetal calf serum (FCS) [17]. Dissociated cells in suspension were passed through a 70-µm strainer and centrifuged at 500 × *g* for 5 min at 4 °C. Red blood cell lysis (ThermoFisher 00-4333-57, Waltham, MA) was done for 2 min and stopped with 10% FCS in PBS. After another centrifugation for 5 min at 500 × *g* (4 °C), the cells were counted using a Neubauer chamber and critically assessed for single-cell separation and viability.

### Immunohistochemistry

C57BL/6NTac mice received either 0 Gy or 18 Gy irradiation to the thoracic cavity. Thoracic-irradiated mice were sacrificed when moribund or at day 130 post-irradiation. The lungs were inflated by intracheal injections of 1 ml of optimal cutting temperature (OCT) (ThermoFisher, Waltham, MA), frozen in −80 °C hexane, sectioned and immunochemistry was performed using antibodies for Fgr (Santa Cruz Biotechnology, Inc # sc-74542) and collagen I (Abcam, ab21286). Five randomly selected images were captured in a blinded fashion from each section using fluorescent confocal microscopy.

### Analysis of lung fibrosis using modified Ashcroft scale

Mice were sacrificed 130 days after 18 Gy thoracic irradiation. The lungs were expsosed and the lungs inflated by intratracheal injection of 1 ml of phosphate-buffered saline and fixed with 10% paraformaldehyde. After paraffin embedding, 4 µm sections were cut from the lungs of the mice. We used 10 mice per condition (control, thoracic-irradiated and thoracic-irradiated and treated with TL02-59). The sections were stained with hematoxylin-eosin (H&E). The validated semiquantitative modified Ashcroft score was used to score pulmonary fibrosis [18, 45]. In short, upon 100× magnification, each successive field was given a score ranging from 0 (normal lung) to 8 (total fibrous obliteration of the field). All scores from the sections were averaged.

### Analysis of collagen content from Masson’s Trichrome staining

Bright-field photomicrographs of Masson’s Trichrome stained tissues were taken in high resolution from randomly selected areas without any selection using a Nikon Eclipse widefield microscope. The images were then quantified for blue trichrome staining using thresholding in ImageJ software (NIH).

### Protein determination by cytokine array

Primary lung fibroblasts were irradiated (5 Gy) and 10 days later, senescence was confirmed by SA-β-GAL staining and morphology. At day 10, the non-irradiated and irradiated cells were counted, and 3 × 10^5^ cells were freshly plated with new media either with or without Fgr inhibitor TL02-59 (10 nM). After 72 h of culture, the media was collected and assayed by cytokine array. Cytokine array was also performed using lung tissue lysates from control (0 Gy), thoracic-irradiated (18 Gy at day 130), and thoracic-irradiated, then TL02-59 treated (10 mg/kg at day 50 for three times a week for 4 weeks) mice. The culture medium was from control non-irradiated mice, FACS isolated radiation-induced senescent (tdTOMp16+), and irradiated and non-senescent cells. To measure the relative levels of each of 62 cytokines/chemokines we used Mouse Cytokine Arrays C3 kit purchased from RayBiotech, (Norcross, GA) and followed manufacturer’s protocol. Briefly, the membranes were blocked for 30 min and incubated with 1 ml of diluted samples (for lung tissue lysates) or undiluted samples (for medium from cultured cells) overnight at 4 °C. The membranes were washed, incubated with Biotinylated Antibody Cocktail, washed, incubated with HRP-Streptavidin, washed, and then developed using the detection buffer and imaged. We used the densitometry software ImageJ (NIH) to obtain spot signal densities from the scanned images. We used an Excel-based Analysis Software tool from RayBiotech for each array kit for automatic analysis.

### BAL fluid analyses

The mice were sacrificed at their experimental endpoints at day 130 post-irradiation, and the lungs were lavaged with 1 ml of DMEM media. In all the mice, the recovery volume was >0.7 ml. BAL fluid was centrifuged 400 × *g* for 10 min at 4 °C, and processed for either RNA isolation, or cytospin slides were prepared by fixing the cells in 4% PFA and using a Shandon Cytospin® 3 Cytocentrifuge (Shandon, Astmoore, UK). RNA samples were processed for cDNA synthesis and slides were processed for immunostaining of BAL cells [46].

### Statistics

For the RT-qPCR analysis of gene expression we used one-way ANOVA followed by post hoc *t* tests. For the other two group comparisons, we used two-sample *t* tests or Wilcoxon rank-sum tests, where appropriate. For the analyses of fibrosis, we used ANOVA followed by Tukey’s multiple comparison tests. *P* values <0.05 were regarded as significant. In these exploratory analyses, we did not adjust *P* values for multiple comparisons.

## Supplementary information

Supplemental Material

## Data Availability

All data are available in the main text or the supplementary materials. RNA-seq and Single-cell RNA sequencing data in the supplement, that support the findings of this study have been deposited in SRA with the accession code PRJNA768885 and PRJNA768942.
