# Supplementary material for: Inhibition of tyrosine kinase Fgr prevents radiation-induced pulmonary fibrosis (RIPF)
Source: Cell Death Discov. 2023 Jul 17;9:252. doi: 10.1038/s41420-023-01538-3 (PMC10352363; doi:10.1038/s41420-023-01538-3)
Supplement: Supplementary file 1 — Supplemental Material [file 41420_2023_1538_MOESM1_ESM.pdf]

Supplemental Figure 1: Induction of gene transcripts shown by heatmap and by analysis of enrichment analyses of chemokine genes from RNAseq in irradiated senescent cells. (A) Heatmap showing chemokine genes in our RNAseq experiment [13] performed from FACS isolated irradiated (5 Gy, 10 days) senescent, irradiated non-senescent and non-irradiated cells in triplicate, from left to right. Heatmap showing top 10 chemokine genes are induced in irradiated senescent cells relative to irradiated non-senescent and non-irradiated cells. 4 genes were upregulated both in irradiated senescent and irradiated non-senescent cells compared to non-irradiated cells. **(B)** GO Enrichment analysis showing chemokine genes were enriched in irradiated senescent cells relative to irradiated non-senescent cells and **(C)** irradiated non-senescent cells and non-irradiated cells.

Supplemental Figure 2. Fgr inhibitor TL02-59 reduces chemokines in SASP from irradiated senescent cells *in vitro*. tdTOMp16+ bone marrow stromal cells were irradiated (5 Gy) and after 10 days tdTOM+ red senescent cells and tdTOM- non-red non-senescent were FACS purified and cultured with or without TL02-59 (10 nM) for 72 hours. Using chemokine array relative amounts of chemokines secreted in the media was measured. (n=2, *, $p<0.05$, p values were calculated using unpaired t-test.

Supplemental Figure 3. Radiation-induced senescence in explanted primary lung fibroblasts. (A) primary fibroblasts were isolated and established from C57BL/6 lungs, irradiated (5 Gy) on culture plates at 50% confluency and 10 days later the cells were stained for senescence using SA-beta-Gal staining kit. **(B)** The number of blue

senescent cells were counted from 3 individual wells and 12% of all cells were senescent at day 10. (n=3, P values were calculated by non-parametric t-test. **(C)** The fibroblasts were stained with phalloidin (FITC) to confirm the presence of actin stress fiber.

Supplemental Figure 4: Detection of Fgr in human fibrotic lungs. Single-cell data from the published IPF atlas was analyzed for Fgr expression, **(A)** In interstitial lung disease patients (ILD) Fgr expression was significantly higher compared to donor controls in monocytes, dendritic cells and mast cells. **(B)** In idiopathic lung disease (ILD) Fgr expression was also significantly higher compared to donor controls in monocytes, macrophages, and dendritic cells **(C)** Surgically resected human RIPF lungs and control non-fibrotic donor lungs were sectioned (4uM) and stained with Masson's Trichrome to confirm severe fibrosis.

Figure S1

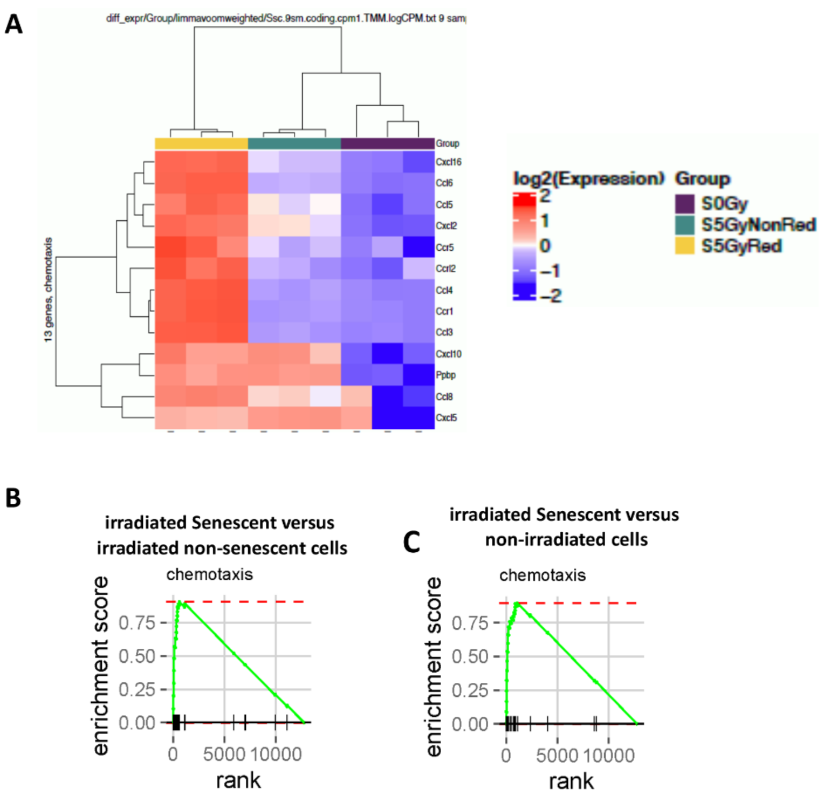
A
diff_expr/Group/limmavoomweighted/Ssc.9sm.coding.cpm1.TMM.logCPM.txt 9 sam
Group
Cxcl16
Ccl6
Ccl5
Cxcl2
Ccr5
Ccrl2
Ccl4
Ccr1
Ccl3
Cxcl10
Ppbp
Ccl8
Cxcl5
13 genes, chemotaxis
log2(Expression)
2
1
0
−1
−2
Group
S0Gy
S5GyNonRed
S5GyRed
B
irradiated Senescent versus
irradiated non-senescent cells
chemotaxis
enrichment score
0.75
0.50
0.25
0.00
0
5000 10000
rank
C
irradiated Senescent versus
non-irradiated cells
chemotaxis
enrichment score
0.75
0.50
0.25
0.00
0
5000 10000
rank

Figure S2

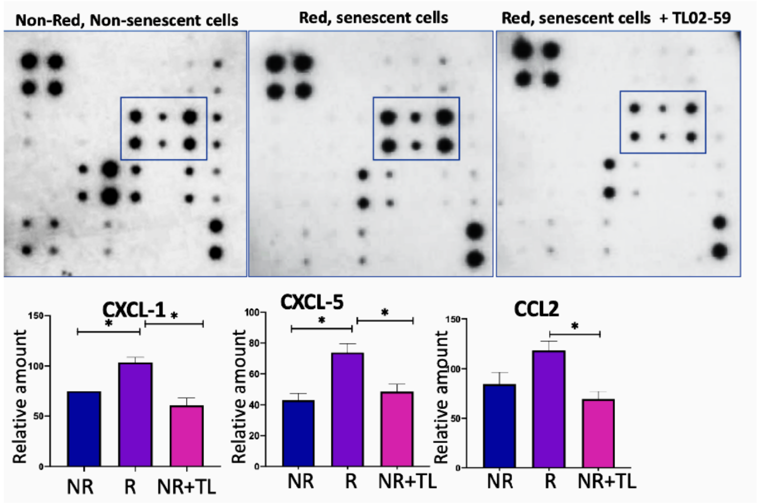

Non-Red, Non-senescent cells
Red, senescent cells
Red, senescent cells + TL02-59
CXCL-1
CXCL-5
CCL2
Relative amount
NR
R
NR+TL

Figure S3

A.

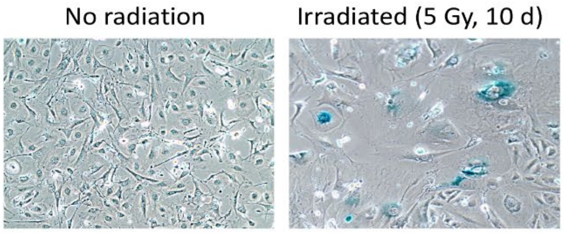

B.

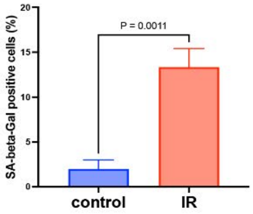

C.

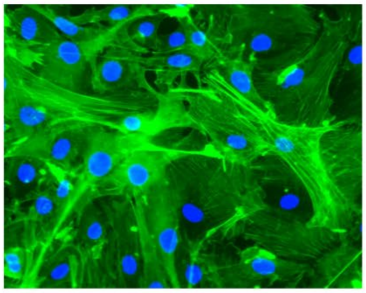

Figure S4

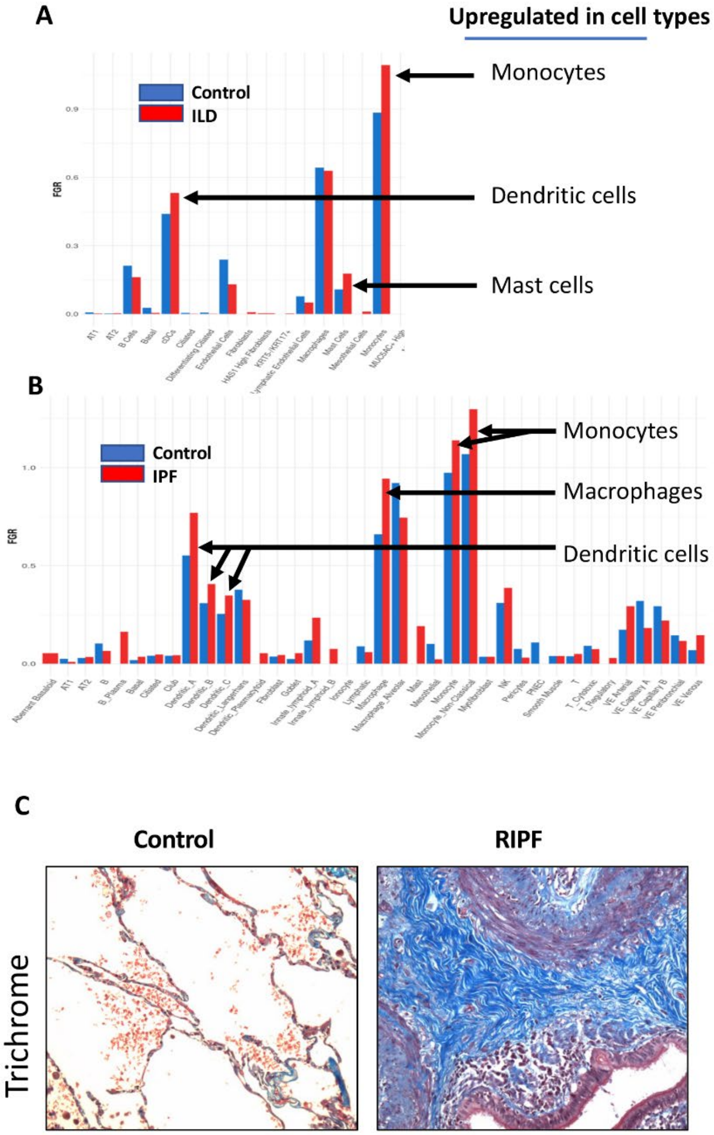
A
Upregulated in cell types
Control
ILD
FGR
Monocytes
Dendritic cells
Mast cells
B
Control
IPF
FGR
Monocytes
Macrophages
Dendritic cells
C
Control
RIPF
Trichrome
